# Inflammatory Myopathy-Related Interstitial Lung Disease: From Pathophysiology to Treatment

**DOI:** 10.3389/fmed.2019.00326

**Published:** 2020-01-17

**Authors:** Baptiste Hervier, Yurdagül Uzunhan

**Affiliations:** ^1^Internal Medicine and Clinical Immunology Department, French Referral Centre for Rare Neuromuscular Disorders, Hôpital Pitié-Salpêtrière, APHP, Paris, France; ^2^INSERM UMR-S 1135, CIMI-Paris, UPMC & Sorbonne Université, Paris, France; ^3^Pneumology Department, Reference Center for Rare Pulmonary Diseases, Hôpital Avicenne, APHP, Bobigny, France; ^4^INSERM UMR1272, Université Paris 13, Bobigny, France

**Keywords:** inflammatory myopathy, myositis, interstitial lung disease, auto-immunity, antisynthetase, anti-MDA-5 autoantibody

## Abstract

Inflammatory myopathies (IM) are auto-immune connective tissue diseases characterized by muscle involvement and by extramuscular manifestations. As such, pulmonary manifestations, which mainly include interstitial lung disease (ILD), often darken two out of four distinct IM, namely dermatomyositis and overlapping myositis. Being the initiation site of the disease and being the leading cause of morbidity and mortality, ILD is of major importance in this context. ILD has a heterogeneous expression among the patients, with various onset mode, various radiological pattern, various severity and finally with different prognoses, which are particularly difficult to predict at the time of IM diagnosis. Therefore, ILD is a challenging issue. Treatments are based on steroids and immunosuppressive or targeted therapies. Their respective place is yet poorly codified however and remains often based on clinician expertise. Dedicated clinical trials are lacking to date and are also difficult to build, due to difficulty of constituting large and homogeneous patient groups and to rigorously evaluate disease outcomes. Indeed, pulmonary function tests alone are being regularly defeated in IM, in which respiratory muscles are often involved. Composite scores, bringing together several lung parameters, should thus be developed and validated in the future, to better assess the disease response to treatment. This review aims to describe the current knowledge of IM immuno-pathogenesis, the clinical features associated with IM related-ILD, focusing of both severity and prognosis, and the actual therapeutic approaches.

## Introduction

Interstitial lung disease (ILD) and inflammatory myopathy (IM) are intimately (1). Diagnosing ILD in patients with IM is associated with worse morbidity and higher mortality than in patients without and therefore conditions the strength of the treatments (2).

In contrast, diagnosing autoimmune features in patients with ILD is of importance, as it confers a better prognosis than idiopathic forms: ILD with autoimmune features but without classification criteria for connective tissue diseases (CTD) as well as connective tissue disease (CTD)-related ILD have a better prognosis than idiopathic ILD (3–5).

**Figure 1:**
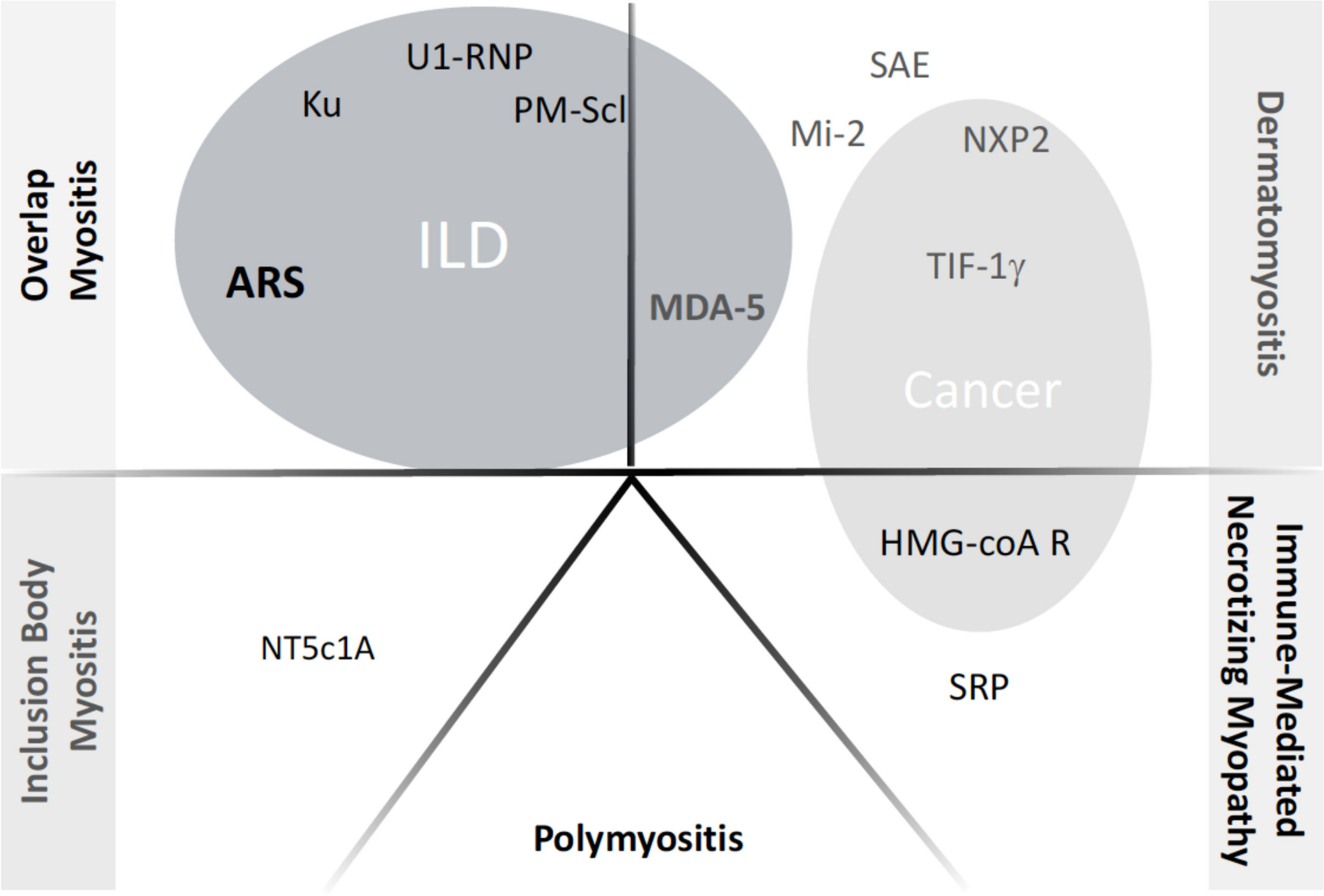
Current classification of inflammatory myopathies and the respective autoantibodies. ILD, Interstitial Lung Disease; ARS, anti-tRNA-antisynthetase autoantidodies, including anti-Jo-1, PL7, PL12, OJ, EJ, Zo, KS, YRS. NXP2: example of myositis specific autoantibody or myositis associated autoantibody; when appearing inside gray circles, the autoantibodies have been shown to correlate with occurrence of either ILD or Cancer, respectively.

The description of IMs has largely evolved over the past decades (6, 7). Based on clinical, immunological and histological features, five groups can be distinguished to date (Figure 1): (i) overlap myositis, which is the most common (ii) dermatomyositis, which often associates a specific skin involvement, (iii) immune mediated necrotizing myopathy (iv) sporadic inclusion body myositis and (v) polymyositis (8–11). These three latter are most of the time restricted to the muscles. The occurrence of ILD is more strongly associated with two out of five IM subtypes (Figure 1). As such, ILD commonly occurs in overlap myositis, among which the anti-synthetase syndrome (aSyS) is the most frequent and can be individualized in many ways (10–14). Other overlap disorders, with myositis-associated autoantibodies (anti-PM-Scl, anti-RNP, anti-Ku etc.) also belongs to this IM subgroup. It is considered that ¾ of the patients with aSyS present with an ILD, whereas this proportion is nearly 1/3 for the other overlap disorders (Table 1). Importantly, ILD may be associated with some phenotype of DM, especially the hypo- or amyopathic forms that are associated with anti-MDA-5 (melanoma differentiation-associated protein 5) auto-antibodies, in which its prevalence reaches up to 90% (15, 24, 25) especially in Asian populations. In association with anti-MDA-5, two distinct types of ILD may be distinguished: the rapidly progressive ILD *vs*. the chronic ILD. In all other cases, ILD occurs more seldom (<10% of the cases) and is most of the time non-severe (26–30).

**Table 1:** Prevalence of ILD in the context of Inflammatory-myopathy.

**Diseases**	**Autoantibodies**	**Prevalence of the ILD**	**References**
	**Myositis-specific autoantibodies**		
Dermatomyositis	MDA-5	90%	(15–17)
Antisynthetase syndrome	All ARS	80%	(18, 19)
	Jo-1	70%	
	Non-Jo-1	85%	
	**Myositis-associated autoantibodies**	
Overlap myositis	RNP	50%	(20)
	PM-Scl	25%	(21, 22)
	Ku	35%	(23)

*ARS, anti-ARNt synthetase auto-antibodies; ILD, Interstitial Lung Disease*.

However, classifying the patients as IM-related ILD is still difficult do date. Indeed, the EULAR-ACR classification criteria for adult and juvenile IM has just been validated, but has many limitations (8, 31, 32). Hence, these classification criteria do not take into account lung involvement and many myositis specific antibodies (MSA). Some patients could thus be misclassified, especially those that are hypo- or amyopathic. Hence, some could classify the patients with ILD, MSA and an hypo- or amyopathic disease as interstitial pneumonia with auto-immune features (IPAF) (33). Obviously, IPAF must not yet be considered as a diagnosis at all, and IPAF classification criteria remain controversial and need to be better defined (34). For instance, some series reported MSA in more than 30% of patients with ILD (35). It is however worth noting that considering IM-related-ILD diagnosis is in fact very important to drive pulmonary and extra-pulmonary management of the patients. Indeed, IM-related ILD has a heterogeneous spectrum, regarding the clinical and radiological features. In the absence of robust markers, prognosis is difficult to predict at diagnosis. Treatments are not standardized, as they have not yet been evaluated rigorously.

By focusing on the two main entities, aSyS and anti-MDA-5 dermatomyositis, the purpose of this review is to describe their immunopathogenesis, the means of assessing ILD activity and progression, as well as severity and prognosis, in order to provide insight into current and future treatments.

## Immunopathogenesis

Autoimmune diseases are multifactorial diseases, tolerance breakdown being the results of various genetic susceptibilities, endocrinal and environmental factors that affect both the innate and adaptive immune system. As one of the largest areas of exchange of the individual with the elements of the environment, some hypothesize that the lungs could be the initiation site of different IMs.

MDA-5 is a protein which functions as an intracellular pattern recognition receptor, recognizing double-strand RNA as danger signals. Upon activation, MDA-5 drives the production of large amounts of type I interferons (36). Anti-MDA-5 dermatomyositis is indeed associated with large amounts of type I interferons and mimics some monogenic interferonopathies (37, 38). However, the reasons leading to type I interferon pathway activation remains unknown to date and anti-MDA-5 autoantibodies have not been demonstrated as being pathogenic. Very little is known regarding the causes and consequences of such direct activation on lung parenchyma and on innate and adaptative immunity, but different data argue for the involvement of macrophages, as it has been reported in various autoimmune diseases (39). These cells may play important roles in immune-regulation and tissue-repair. As such, recent data have revealed that non-inflammatory macrophages (previously called M2 macrophages, which produce IL-10 and TGFβ) are involved in the progression of lung fibrosis (40, 41). Interestingly, soluble macrophage-mannose receptor, sCD206, a serum marker for M2 polarization, is increased in MDA-5 DM-associated ILD and its titer correlated with a poor outcome (42). Interleukin(IL)-18 (43), a potent macrophage activating molecule could be involved in the development of ILD. In addition, several macrophage activation markers, including ferritin (44), NOS2 or neopterin are increased in the patients with anti-MDA-5 dermatomyositis.

ASyS is a heterogeneous disease, immunologically characterized by MSA directed against different ARNt-synthetases, among which anti-hystidyl-tRNA-synthetase (also called anti-Jo-1) is the most common (18, 45). To date, seven auto-antibodies directed against other tRNAsynthetases, including anti-Alanyl (PL-12), anti Threonyl (PL-7), anti-Glycyl (EJ) -t-RNA-synthetases have been described. Although dark areas persist, the immunopathogenesis of Jo-1 positive aSyS is best described and could nowadays be drawn as follows: following environmental exposure to tabacco smoke (46), airborne contaminants (47) including mineral particles (such as asbestiform amphiboles) or respiratory tract infections (48), the lung tissue is aggressed. This leads to cellular stress, danger signal pathway activation and cell death with microparticle release. Innate immune cells–such as NK cells- are unspecifically activated and release proteolytic enzymes, including Granzyme B (49). The antigen, Histidyl-tRNA-synthetase, which is expressed into a specific conformation within the lungs, is then released in the extracellular milieu and has many immune properties, including activity in inflammatory response with its cytokine like domains, chemoattractant properties with CCR5^+^ cell recruitment and capacity to activate other immune cells (50, 51). All the immune cells are present within the lungs of patients with aSyS (52). Tolerance breakdown may occur when the different adaptive immune cells are successively activated. The cascade of events is efficiently favored by a certain genetic background, like HLA-B^*^08.01 (53), and includes antigen presentation, CD8-T cell priming and CD4-T cell-B-cell crosstalk. As a witness of these processes, type I/II interferons, B lymphocyte stimulator and other cytokines are increased in the sera of aSyS patients. Finally, anti-Histidyl-tRNA-synthetase autoantibodies are produced. The way the disease propagates to other organs remains largely unexplained: although histydyl-tRNA-synthetase could be abnormally expressed in various tissues, the pathogenicity of anti-Jo-1 is still matter of controversies and the presence of Jo-1 specific T cells within extra-pulmonary target tissues has to be further determined.

## Initial Evaluation

### Clinical Evaluation of the ILD

Patients with IM-related ILD may present with clinical symptoms, including fever (1/4), cough (1/3) or dyspnea (>1/2), which could be either related to ILD or not, especially when gastro-esophageal reflux or respiratory muscle involvement also occurs as part of the aSyS (18, 19). Regarding the shortness of breath, it is immediately important to evaluate (i) the rapidity of onset, as the (sub)acute forms settling within 3 months –defining rapidly-progressive (RP)-ILD are of worse prognosis (54, 55) and (ii) the severity, as some patients require intensive care support (56). In contrast, the patients with mild ILD or in which ILD will develop later in the follow-up (1/5) can be asymptomatic, justifying careful explorations.

### Explorations of the ILD

The severity of ILD can also be assessed by oximetry and blood gases to evaluate hypoxemia and/or hypercapnia.

**Figure 2:**
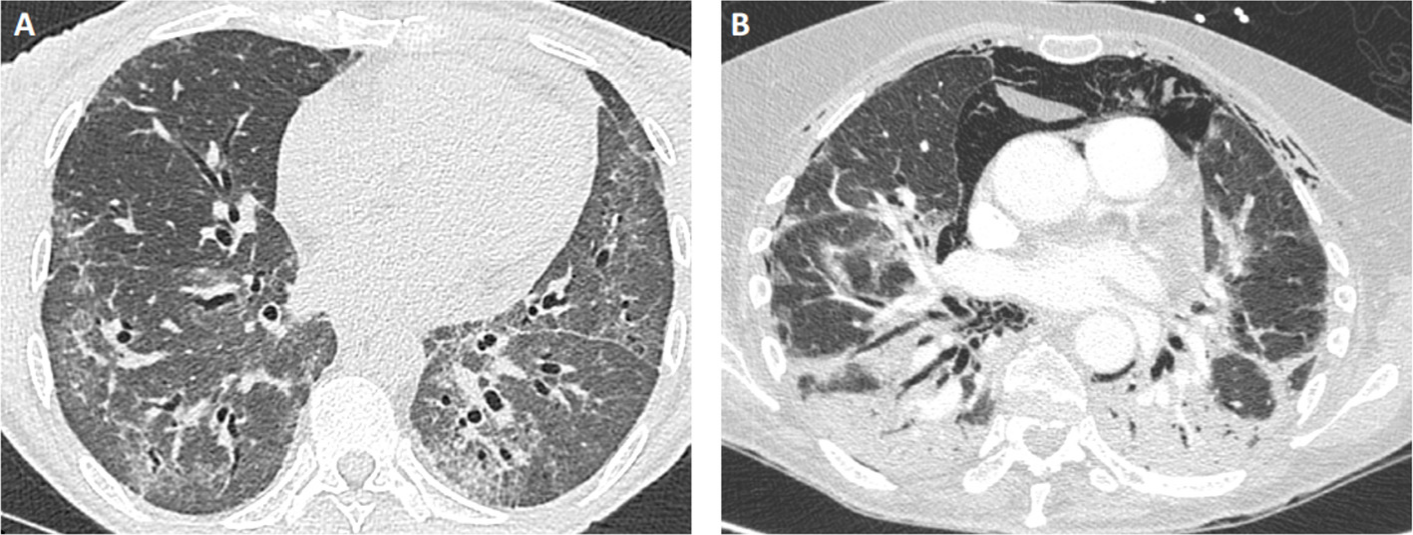
Examples of lung CT findings in patients with IM-related ILD. **(A)** NSIP pattern in a patient with PL12+ antisynthetase syndrome: ground glass opacities with bilateral proximal bronchiectasis. **(B)** OP pattern in a patient with MDA5+ dermatomyositis: extensive parenchymal consolidation and pneumomediastinum.

CT-Scan is the major tool of the evaluation (Figure 2), revealing different types of lesions and helping in classifying ILD into different patterns, as defined by the ATS/ERS consensus for idiopathic interstitial pneumonias (Table 2) (61). As such, bi-basal ground glass opacities and linear reticulations are associated with non-specific interstitial pneumonia (NSIP) and are the most common, as found in other connective tissue disorders like systemic sclerosis. Alveolar condensations -willingly bilateral- also occur, especially in the RP-ILD subset and define organizing pneumonia (OP). Both patterns may also mix together (NSIP-OP). Usual interstitial pneumonia (UIP), with sub-pleural honey combing lesions, is less frequent and dramatically more seldom in IM-related ILD than in rheumatoid arthritis-related ILD. In the worse cases with acute lung injury, often leading to acute respiratory distress syndrome (ARDS), CT-scan may show features of acute interstitial pneumonia with consolidations and extensive ground glass opacities.

**Table 2:** ILD-patterns on lung CT-scan: lesion types and prevalence in IM-related ILD.

**ILD pattern**		**Predominant lesions on CT-scan**	**Prevalence in ASyS**	Prevalence in MDA5^*^
Non-Specific Interstitial Pneumonia	NSIP	Basal ground glass opacities, linear reticulations	50%	20%
Organizing Pneumonia	OP	Alveolar consolidations	20%	50%^**^
	NSIP-OP	Associations of NSIP & OP lesions	25%	30%
Usual Interstitial Pneumonia	UIP	Basal subpleural reticulations with bronchectasis and honeycombing lesions	10%	<5%
Acute Interstitial Pneumonia	AIP	Consolidations and extensive ground-glass opacities	<5%	30%^**^
**Other associated anomalies**				
Signs of Fibrosis		Reticulations, Traction Bronchectasis	>75%	40%
Non-significant adenopathies			30%	30%

* *To be confirmed in larger series*,

** * OP and AIP are often difficult to distinguish. Adapted from (56–60)*.

CT-scan is also important to evaluate (i) the presence of fibrosing lesions, including traction bronchiectasis and reticulations, which are present at first evaluation in high proportion (57, 62), and (ii) the extension of the lesions (63) -usually bilateral and starting in posterior and basal regions- within all the lung parenchyma.

The distribution of these patterns partially depends on the IM subtype, NSIP predominating in aSyS and OP in MDA-5-dermatomyositis (Table 2) (58).

At diagnosis (as well as in any case of worsening during follow-up), endoscopy and broncho-alveolar lavage (BAL) could be discussed. It might help in distinguishing specific deterioration from intercurrent factors that may have caused respiratory decline, including aspiration pneumonia in newly diagnosed patients, or opportunistic infections, which occur mostly in patients under immunosuppressive therapy. In addition, rare cases of bronchiolo-alveolar cancer can be thus detected. BAL fluid discloses aspecific alveolitis with high counts of lymphocytes, neutrophils, eosinophils or with mixed cellularity.

Lung histology is no longer recommended due to the low benefit/risk ratio of the biopsy procedure, and ILD subtype could be almost easily determined on CT-scan rather than on histological features.

When possible, pulmonary function tests help in evaluating ILD severity, as well as detecting a possible respiratory muscle involvement. Restrictive syndrome, defined by a total lung capacity (TLC) <80% with a more or less severe decrease of forced vital capacity (FVC), is almost constantly observed. Severity of restriction may be appreciated by TLC impairment on plethysmography. However, the FVC impairment is more routinely followed as it is more easily measured on spirometry. Muscle impairment and especially diaphragmatic involvement may worsen this parameter and is suggested when FVC is dramatically lower than expected as compared to CT-scan lesions or when decubitus FVC is significantly lower than conventional FVC. Diffusing capacity of the lung for carbon monoxide (DLCO) impairment often precede TLC and FVC decrease. DLCO is also useful to evaluate the ILD severity at any time of the disease course and/or to suspect pulmonary hypertension when excessively reduced in comparison to FVC deterioration. In this context, the screening for pulmonary hypertension by trans-thoracic echocardiography is recommended (64).

Exploring diaphragmatic involvement might be useful. Measurement of maximal static inspiratory pressure (PI max) and of maximal static expiratory pressure (PE max) are low invasive parameters that could be combined with radioscopic assessment of diaphragmatic course and electromyography of the diaphragm to explore significant diaphragmatic dysfunction (65, 66).

Six-minutes walk test (6MWT) may be useful to estimate ILD severity in the absence of significant muscle involvement. Dyspnea, nadir of oxygen saturation and walking distance are the main parameters evaluated during the test.

FVC, DLCO and in a lesser extend 6MWT are therefore main physiologic parameters for assessing respiratory severity at diagnosis and also to evaluate response to therapy during the disease course.

### Extra-Pulmonary Evaluation

Besides pulmonary evaluation, muscle, skin, heart, joint, and vessel involvement must be carefully assessed. Above all, severe myositis with dysphagia and respiratory muscle involvement may complicate the management of ILD and biased the ILD evaluation.

A particular attention should be paid for skin manifestations, as ulcerations are found especially in MDA-5+ dermatomyositis (16).

In all patients with newly identified ILD, the last ATS/ERS/ALAT/JRS recommendations indicated an overwhelming agreement to perform serological testing to achieve a diagnosis rigorously. The majority of panelists acknowledged routine testing for C-reactive protein, antinuclear antibodies, myositis linear-dot panel or immunoprecipitation, as well as rheumatoid factor and anti–cyclic citrullinated peptide for Rheumatoid arthritis (67). Other detailed tests, such as creatine kinase, have to be performed on a case-by-case basis according to the associated clinical signs.

## Evolution

### Short-Term Prognosis

Three factors, that are linked together, could be identified as short-term prognosis factors: (i) the severity of the ILD itself, (ii) the rapidity of onset (RP-ILD) (54, 55) and (iii) the presence of anti-MDA-5 auto-antibodies (15). Even in the absence of any comparative study, it is admitted that the most severe patients with either aSyS or anti-MDA-5 dermatomyositis, notably those requiring intensive care, are of worse prognosis. In these patients, the severity is evaluated clinically or with the CT-scan (showing extended OP or acute interstitial pneumonia lesions), but rarely with the pulmonary function tests, often impossible to perform. In intensive care unit, the mortality ratio reaches 50% (56). Most of the severe patients presented a RP-ILD, which is itself associated with a high mortality risk ratio, as compared to patients with chronic onset of ILD, both during aSyS and anti-MDA-5 dermatomyositis. When comparing patients according to the nature of the myositis specific autoantibodies, it has been clearly shown that the presence of MDA-5 autoantibodies is by itself a risk factor of early mortality, as compared to anti-ARNt-synthetases (15).

### Long-Term Prognosis

#### General Outcome

Despite early mortality in the severe forms, the 5-year survival ratio is >85% in IM-ILD (18, 19, 59). Although some patients could worsen during the first year of treatment, the time to disease progression usually counts in years (57, 58). As examples, in long-term follow-up series, 20% of the patients with IM-related ILD (not including patients with anti-MDA-5) worsen despite immunosuppressive treatments, with the risk of developing respiratory failure. The remaining patients being stable (35–55%) or improved (25–45%) (59, 68, 69). It is thus important to find factors predictive of ILD progression over time, especially during the first months of treatment. However, the heterogeneity of IM-related ILD makes assessment of prognosis particularly difficult, not allowing us to clearly stratify the patient and adjust the treatment to the potential of aggravation. Such attitude is still a real challenge and should be the subject of future studies.

#### Evaluations

The severity of the ILD on pulmonary functions tests is probably not sufficient to predict long-term evolution. Some retrospective studies suggested a correlation between the PFTs at onset (such as low DLCO or FVC) and the long-term ILD prognosis (59, 70). However, in a prospective cohort, the first value of either FVC or DLCO did not correlate with improvement or worsening over time (71). This was at least partly due to the existence of respiratory muscle involvement, which is a confusing factor to interpret FVC as a marker of lung involvement only. It could be thus more relevant to evaluate the kinetic of FVC variations between two early time points, as a predictive factor of long-term response to treatment. However, such option has not been validated prospectively in large cohorts.

It has been demonstrated in studies dedicated to IPF, that serial decline in the FVC over 6–12 months is a powerful predictor of mortality (72). An absolute change in the FVC of 10% of the predicted normal value is a predictor of mortality but this large amplitude of change is less prevalent than relative change in a given time period, which has been shown to be also predictive of mortality in the majority of IPF studies. More recently, Goh et al. (73) have examined correlations between short-term pulmonary function trends and long-term outcome in ILD associated with systemic sclerosis, which is very close to chronical forms of IM-related ILD. Disease severity at baseline and subsequent pulmonary function trends were independent prognostic determinants. At 1 year, categorical FVC trends provide the most accurate prognostic information, especially when integrated with DLCO trends. Thus, the optimal definition of categorical decline in FVC for trial purposes may consist of either a ≥10% decline in the FVC or a 5–9% marginal decline in the FVC in association with a ≥15% decline in the DLCO for systemic sclerosis. However, such studies are lacking in IM-related ILD (74).

Thus, as suggested by these studies, it would be probably more accurate in IM-related ILD also to at least consider these parameters as qualitative variable, taking into account the proportion of patients improving/worsening FVC and/or DLCO for at least 10 and 15%, respectively.

Furthermore, defining time to ILD progression or event-free survival could be relevant and should be rigorously evaluated in the future as end-points. Composite scores including dyspnea score, muscle and physiologic parameters would probably be of great interest for evaluating disease progression and treatment response in IM-related ILD. Defining and validating such scores will be a challenge in the future.

Valuable information coming from CT-scan analyses is also insufficiently robust to date. Regarding the ILD radiologic pattern, some suggested UIP was worse than NSIP, especially in terms of disease progression (68). Although histology of UIP is more frequent than expected in the autopsy series, the corresponding radiologic pattern has however a better prognosis than IPF (75). A recent large study showed that the UIP pattern on lung-CT-scan is significantly associated with mortality. As opposed to acute interstitial pneumonia, the OP pattern was associated with the lowest mortality on long-term follow-up (76). No study has demonstrated yet a worse prognosis according to either fibrosing scores and/or extension scores assessed on CT-scan in IM-related ILD, as it has been for example reported in systemic sclerosis (63). At least, anti-rheumatic drug modifications (DMARDs) overtime correlated with the initial extension of the ILD within the lung parenchyma (60).

Different biomarkers could correlate with ILD prognosis. However, further studies are required to validate on a large scale the promising interest of KL-6 (77), Ferritin (44), C-RP or IL-18 serum dosage (43) alone or mixed together, to perform them routinely and stratify the patient with IM-related ILD early, according to their potential prognosis value. In ASyS patients, it has been shown that patients with non-Jo-1 had a worse prognosis in terms of mortality as compared to Jo-1 patients (18, 19). Although not rigorously demonstrated, these data could be at least due to higher proportions of hypo- or amyopathic patients in the non-Jo1 group, in which the ILD could therefore be more severe upon diagnosis. The concomitant positivity of anti-Ro 52 kilo-daltons, which is quite common in IM and especially in ASyS (78) might worsens the ILD prognosis (79). Using unsupervised analyses, three distinct subgroups with different prognoses can be observed on a large French multicentric cohort of MDA-5 dermatomyositis (Allenbach et al. unpublished data). The first cluster with severe lung involvement and a dramatically poor prognosis corresponded to the well-recognized “anti-MDA5+ RP-ILD.” In addition, two other overlapping forms were isolated: the “anti-MDA5+ arthro-DM,” with a good prognosis, and the “anti-MDA5+ vasculo-DM,” with an intermediate prognosis. The decisional algorithm showed that only three variables (Raynaud phenomenon, arthralgia/arthritis and gender) are good predictors for cluster appurtenance and their related outcome.

### ILD Complications

Besides progression of fibrosing ILD, aspiration or opportunistic pneumonia, IM-related ILD has two major complications.

Although rare (<8% of the cases) and often associated with pneumothorax, pneumomediastinum is non-fortuitously associated with IM-related ILD, as it occurs more commonly than in other connective tissue-related ILD (80). Association with MDA-5 auto-antibodies, long suspected from various reports (81–83) has been rigorously demonstrated only recently (84). Pneumomediastinum is an aggravating factor, that usually occurs early (<24 months) in the course of the disease. Its underlying mechanism remains unknown to date.

Pulmonary hypertension is the second feared ILD complication. In contrast to pneumomediastinum, pulmonary hypertension occurs lately in the course of ILD and witnesses its severity. Indeed, during aSyS and conversely to other connective tissue diseases, pulmonary hypertension belongs to group III only and is diagnosed in almost 8% of the cases (64). However, in the severe forms, a contribution of a vascular component is not excluded. Even though there is no recommendation for pulmonary hypertension specific treatments in this context, its screening with repeated echocardiography is recommended. When necessary, right heart catheterization will confirm the diagnosis. In a French series, patients with pulmonary hypertension had a significantly lower survival rate.

Thus, finding efficient prognosis factors (or prognosis scores pooling the different parameters), correlating with long-term disease severity is of major importance, and should be the prospect of future studies. Indeed, the development of patient stratification according to the risk of progression, in order to manage therapeutic strategies for each patient. Such personalized medicine remains a challenge in the field of IM-related ILD.

## Treatments

### Adjuvant Therapies

Besides different possibilities of medical treatment, patients with IM-related ILD must benefit from the update of the vaccines, like annual vaccination against flu, anti- pneumococcal vaccination. Such attitude is indeed justified by a recent study showing that antibody response rates in the connective tissue-related ILD patients (including those receiving immunosuppressants) were comparable with those of a control group without ILD (85). In addition, no acute exacerbation was observed after pneumococcal immunization, indicating pneumococcal vaccines in ILD patients are efficient and safe (85).

Occurrence of opportunistic infections in IM-related ILD is significant and could be at least associated with the disease itself and its treatments (86). Thus, preventive treatment of *pneumocystis jirovecii* with trimethoprime + sulfamethoxazole or in case of contraindication with atovaquone, should be prescribed as soon as patients received steroids >20 mg/d during >4 weeks and especially for the most severe patients (87).

Pulmonary rehabilitation as well as muscle physiotherapy may also be beneficial (88). Since nutrition-related factors have been noticed as a prognostic factor for patients with chronic respiratory diseases, including patients with ILD, particular attention should also be paid to this aspect of the patients' care (89). When clearly implicated and if possible, exposure to cigarette smoke and other airborne contaminants should be avoided.

All patients should benefit from this personalized treatment approach. Therapeutic education programs should address symptom management, oxygen therapy and medications. Patients emphasized the importance of understanding what the future might hold and were generally supportive of discussing advance care planning and end-of-life care.

### Steroids and Classical Immunosuppressive Drugs

In the absence of randomized clinical trials, treatments of IM-related LD are based on small retrospective studies. Treatment efficacy is difficult to evaluate in this context and requires sufficiently long evaluation period. In most of the studies, the outcome measures are improvement of pulmonary function tests between two time points (FVC and/or DCLO being considered as quantitative variables). However, FVC also depends on respiratory muscle involvement and make respiratory evaluations difficult when the IM is severe.

Even though we noticed the absence of dedicated trial, treatment of IM-related ILD is based on steroids. Intravenous high doses are initially given in the most severe forms or RP-ILD.

Addition of an immunosuppressive drug as a first line treatment progressively became consensual, being now a cornerstone of the treatment, as ¾ of the patients could develop steroid resistance or relapse when tapering the doses (90), irrespectively of the initial severity. As such, cyclophosphamide and tacrolimus have been reported in retrospective studies to improve FVC and/or DLCO in almost all patients (91–93). Although less commonly reported, azathioprine and methotrexate could also be efficient (94, 95). Interestingly, tacrolimus and mycophenolate mophetil have shown interest in reducing steroid doses. Recently, one study has compared aztioprine vs. mycophenolate mophetil: both improved PFTs in similar proportions (96). Azathioprine allowed a greater decrease in the dose of steroids as compared to mycophenolate mophetil, while being associated with more side effects. Among these immunosupressants, intravenous cyclophosphamide, mycophenolate mofetil, and azathioprine have been reported to be efficient in similar proportions (97).

Some reports emphasize the interest of immunosuppressive treatment associations (98), especially when ILD is severe. However, such attitude exposes the patients to higher infectious risks.

IM-related ILD is a chronical disease and requires prolonged treatment duration, often exceeding several years. There is however no clear information to date regarding the most appropriate time and modalities to stop the treatments.

Single case reports indicated some benefit from plasma exchange for IM associated severe ILD, especially those with anti-MDA-5 autoantibodies, but no conclusion could be drawn to date. While some reported its use as an initial treatment in severe ASyS patients (99), no data support the long-term efficacy of intravenous immunoglobulin treatment for ILD in the context of IM.

### Biologics

Over the past decades, the relative place of biologics to treat IM-related ILD has increased a lot. Among them, the anti-CD20 targeting B-cell therapy has become the most documented. In one of the few prospective studies, 50% of refractory AsyS patients receiving rituximab as a third line therapy improved their FVC at 1 year (100). Several retrospective studies (101–103) and a meta-analysis (104) reported promising results of rituximab on pulmonary function tests. On the other hand, efficacy of rituximab based on the improvement of CT-score was less clear. However, the cost in terms of risk of infections, with sometimes fatal complications, is high (101). In these retrospective series, rituximab was most of the time used as at least a second line treatment and there was no comparison with other treatments. Thus, the place of anti-CD20 monoclonal antibodies in the therapeutic arsenal needs further clarifications, which will emerge from prospective trials currently in progress.

Other targeted therapies have been tried in severe RP-ILD associated with anti-MDA5 dermatomyositis. On the faith of a small case series of four patients, basiliximab, a monoclonal antibody targeting CD25+ activated T cells, could improve patients' survival (105). Similarly, JAK-inhibitors (in this case tofacitinib), which blocks interferon pathways and other pro-inflammatory cytokine pathways, has shown a promising survival rate improvement (17, 106).

Of note, anti-TNFα targeting therapies are usually not recommended in the context of IM (107), partly due to the occurrence of muscular aggravations under treatment.

### Future Directions

Depending on a better understanding of the immune mechanisms leading to ASyS and MDA-5 dermatomyositis, new immune-based therapeutic strategies could emerge in the future. As such, different existing biologics could find a place to treat IM-related ILD, including anti-IL12/23, anti-IFNα and anti-IFNα receptors antibodies, anti-IL-6 or other anti-B cell therapy like ibrutinib etc. However, the rational to use these treatments lack translational data to date showing a clear involvement of these pathways in ILD pathogenesis. New directions could also be developed in the future according to these immunological researches and help in developing new treatments. As examples, blocking pattern recognition receptor-dependent immune cell activation or macrophage activation pathways, which seems specifically involved in ILD associated with MDA-5 positivity might become real and might open a new era in the future. Future immunotherapies have to integrate innovative approaches based on selective and oriented immunomodulations as well as on concomitant therapies promoting tissue repair. Anti-fibrotic agents could be a new treatment option: (i) fibrotic mechanisms are at work in the lung of patients with IM-related ILD, (ii) the recent results obtained in patients treated with nintedanib for systemic sclerosis-related ILD, another connective tissue disease associated with fibrosing ILD, are promising (108). Although such clinical trials required a large number of patients to be informative, efforts should be done to define eligible patients and to build international and randomized prospective trials, at least in ASyS-related ILD.

### Lung Transplantation

Few cases of lung transplantation have been reported in patients with IM-related ILD (109). Of note, comorbidities as well as immune fragility of the patients, related to the previous immunosuppressive treatment they received, negatively impact the prognosis of the procedure. In addition, involvement of respiratory muscles, especially in ASyS, and/or skin vascular sequelae in MDA-5 positive patients are probably factors of transplantation failure. However, in patients carefully selected the reported risk of IM-related ILD recurrence is not higher than that of other connective tissue disorders, including systemic sclerosis, and a 5-year survival rate of 75% has been described in a small case series (110). Thus, lung transplantation is possible in IM-related ILD and its prognosis factors for success should be more largely studied worldwide. Extracorporeal membrane oxygenation (ECMO) may be interesting as a bridge to lung transplantation in selected patients already considered as candidates for lung transplantation. Thus, referring severe patients to transplantation centers early in the course of the disease is important.

## Conclusion

Although the knowledge of IM-related ILD has tremendously progressed over the past decades, its management remains a challenge to date. Based on basic and clinical research, the future objectives will need to focus on the IM-related ILD definition of classification criteria, the development of reliable disease activity and progression scores that can be used as robust end-point for the future clinical trials and the finding of early prognosis biomarkers. The aims will be to adapt therapeutic strategies to individual risk factors (patients' stratification) and to find new efficient immune-based biologics as well as to prospectively study the relevance of innovative anti-fibrotic agents.

## Author Contributions

BH and YU wrote the manuscript and built the tables and figures, which are original.

### Conflict of Interest

The authors declare that the research was conducted in the absence of any commercial or financial relationships that could be construed as a potential conflict of interest.

## References

[1] Mecoli CA Christopher-Stine L. Management of interstitial lung disease in patients with myositis specific autoantibodies. Curr Rheumatol Rep. (2018) 20:27. 10.1007/s11926-018-0731-7 29637383

[2] Cottin V Thivolet-Béjui F Reynaud-Gaubert M Cadranel J Delaval P Ternamian PJ. Interstitial lung disease in amyopathic dermatomyositis, dermatomyositis and polymyositis. Eur Respir J. (2003) 22:245–50. 10.1183/09031936.03.00026703 12952255

[3] Park JH Kim DS Park I-N Jang SJ Kitaichi M Nicholson AG. Prognosis of fibrotic interstitial pneumonia: idiopathic versus collagen vascular disease-related subtypes. Am J Respir Crit Care Med. (2007) 175:705–11. 10.1164/rccm.200607-912OC 17218621

[4] Navaratnam V Ali N Smith CJP McKeever T Fogarty A Hubbard RB. Does the presence of connective tissue disease modify survival in patients with pulmonary fibrosis? Respir Med. (2011) 105:1925–30. 10.1016/j.rmed.2011.08.015 21924888

[5] Yoshimura K Kono M Enomoto Y Nishimoto K Oyama Y Yasui H. Distinctive characteristics and prognostic significance of interstitial pneumonia with autoimmune features in patients with chronic fibrosing interstitial pneumonia. Respir Med. (2018) 137:167–75. 10.1016/j.rmed.2018.02.024 29605201

[6] Betteridge Z Tansley S Shaddick G Chinoy H Cooper RG New RP. Frequency, mutual exclusivity and clinical associations of myositis autoantibodies in a combined European cohort of idiopathic inflammatory myopathy patients. J Autoimmun. (2019) 101:48–55. 10.1016/j.jaut.2019.04.001 30992170 PMC6580360

[7] Mariampillai K Granger B Amelin D Guiguet M Hachulla E Maurier F. Development of a new classification system for idiopathic inflammatory myopathies based on clinical manifestations and myositis-specific autoantibodies. JAMA Neurol. (2018) 75:1528–37. 10.1001/jamaneurol.2018.2598 30208379 PMC6583199

[8] Lundberg IE Tjärnlund A Bottai M Werth VP Pilkington C de Visser M. 2017 European League Against Rheumatism/American College of Rheumatology classification criteria for adult and juvenile idiopathic inflammatory myopathies and their major subgroups. Ann Rheum Dis. (2017) 76:1955–64. 10.1136/annrheumdis-2017-211468 29079590 PMC5736307

[9] Lilleker JB Vencovsky J Wang G Wedderburn LR Diederichsen LP Schmidt J. Response to: ‘Antisynthetase syndrome or what else? Different perspectives indicate the need for new classification criteria' by Cavagna et al. Ann Rheum Dis. (2018) 77:e51. 29259048 10.1136/annrheumdis-2017-212382

[10] Selva-O'Callaghan A Pinal-Fernandez I Trallero-Araguás E Milisenda JC Grau-Junyent JM Mammen AL. Classification and management of adult inflammatory myopathies. Lancet Neurol. (2018) 17:816–28. 10.1016/S1474-4422(18)30254-0 30129477 PMC11646336

[11] Noguchi E Uruha A Suzuki S Hamanaka K Ohnuki Y Tsugawa J. Skeletal Muscle Involvement in Antisynthetase Syndrome. JAMA Neurol. (2017) 74:992–9. 10.1001/jamaneurol.2017.0934 28586844 PMC5710328

[12] Stenzel W Preuße C Allenbach Y Pehl D Junckerstorff R Heppner FL. Nuclear actin aggregation is a hallmark of anti-synthetase syndrome-induced dysimmune myopathy. Neurol. (2015) 84:1346–54. 10.1212/WNL.0000000000001422 25746564

[13] Aouizerate J De Antonio M Bassez G Gherardi RK Berenbaum F Guillevin L. Myofiber HLA-DR expression is a distinctive biomarker for antisynthetase-associated myopathy. Acta Neuropathol Commun. (2014) 2:154. 10.1186/s40478-014-0154-2 25339355 PMC4210467

[14] Mescam-Mancini L Allenbach Y Hervier B Devilliers H Mariampillay K Dubourg O. Anti-Jo-1 antibody-positive patients show a characteristic necrotizing perifascicular myositis. Brain J Neurol. (2015) 138:2485–92. 10.1093/brain/awv192 26198592

[15] Labrador-Horrillo M Martinez MA Selva-O'Callaghan A Trallero-Araguas E Balada E Vilardell-Tarres M. Anti-MDA5 antibodies in a large Mediterranean population of adults with dermatomyositis. J Immunol Res. (2014) 2014:290797. 10.1155/2014/290797 24741583 PMC3987881

[16] Narang NS Casciola-Rosen L Li S Chung L Fiorentino DF. Cutaneous ulceration in dermatomyositis: association with anti-melanoma differentiation-associated gene 5 antibodies and interstitial lung disease. Arthritis Care Res. (2015) 67:667–72. 10.1002/acr.22498 25331610 PMC4404195

[17] Kurasawa K Arai S Namiki Y Tanaka A Takamura Y Owada T. Tofacitinib for refractory interstitial lung diseases in anti-melanoma differentiation-associated 5 gene antibody-positive dermatomyositis. Rheumatol. (2018) 57:2114–9. 10.1093/rheumatology/key188 30060040

[18] Hervier B Devilliers H Stanciu R Meyer A Uzunhan Y Masseau A. Hierarchical cluster and survival analyses of antisynthetase syndrome: phenotype and outcome are correlated with anti-tRNA synthetase antibody specificity. Autoimmun Rev. (2012) 12:210–7. 10.1016/j.autrev.2012.06.006 22771754

[19] Aggarwal R Cassidy E Fertig N Koontz DC Lucas M Ascherman DP. Patients with non-Jo-1 anti-tRNA-synthetase autoantibodies have worse survival than Jo-1 positive patients. Ann Rheum Dis. (2013) 10.1136/annrheumdis-2012-201800 23422076 PMC4031026

[20] Szodoray P Hajas A Kardos L Dezso B Soos G Zold E. Distinct phenotypes in mixed connective tissue disease: subgroups and survival. Lupus. (2012) 21:1412–22. 10.1177/0961203312456751 22864236

[21] D'Aoust J Hudson M Tatibouet S Wick J Mahler M Baron M. Clinical and serologic correlates of anti-PM/Scl antibodies in systemic sclerosis: a multicenter study of 763 patients. Arthritis Rheumatol. (2014) 66:1608–15. 10.1002/art.38428 24577935

[22] De Lorenzo R Pinal-Fernandez I Huang W Albayda J Tiniakou E Johnson C. Muscular and extramuscular clinical features of patients with anti-PM/Scl autoantibodies. Neurology. (2018) 90:e2068–76. 10.1212/WNL.0000000000005638 29728522 PMC5993182

[23] Rigolet A Musset L Dubourg O Maisonobe T Grenier P Charuel J-L. Inflammatory myopathies with anti-Ku antibodies: a prognosis dependent on associated lung disease. Medicine. (2012) 91:95–102. 10.1097/MD.0b013e31824d9cec 22391471

[24] Moghadam-Kia S Oddis CV Sato S Kuwana M Aggarwal R. Antimelanoma differentiation-associated Gene 5 antibody: expanding the clinical spectrum in North American patients with Dermatomyositis. J Rheumatol. (2017) 44:319–25. 10.3899/jrheum.160682 28089977

[25] Hall JC Casciola-Rosen L Samedy L-A Werner J Owoyemi K Danoff SK. Anti-melanoma differentiation-associated protein 5-associated dermatomyositis: expanding the clinical spectrum. Arthritis Care Res. (2013) 65:1307–15. 10.1002/acr.21992 23436757 PMC3689861

[26] Miller T Al-Lozi MT Lopate G Pestronk A. Myopathy with antibodies to the signal recognition particle: clinical and pathological features. J Neurol Neurosurg Psychiatry. (2002) 73:420–8. 10.1136/jnnp.73.4.420 12235311 PMC1738058

[27] Hengstman GJD Vree Egberts WTM Seelig HP Lundberg IE Moutsopoulos HM Doria A. Clinical characteristics of patients with myositis and autoantibodies to different fragments of the Mi-2 beta antigen. Ann Rheum Dis. (2006) 65:242–5. 10.1136/ard.2005.040717 16410528 PMC1798024

[28] Tarricone E Ghirardello A Rampudda M Bassi N Punzi L Doria A. Anti-SAE antibodies in autoimmune myositis: identification by unlabelled protein immunoprecipitation in an Italian patient cohort. J Immunol Methods. (2012) 384:128–34. 10.1016/j.jim.2012.07.019 22884621

[29] Betteridge ZE Gunawardena H Chinoy H North J Ollier WER Cooper RG. Clinical and human leucocyte antigen class II haplotype associations of autoantibodies to small ubiquitin-like modifier enzyme, a dermatomyositis-specific autoantigen target, in UK Caucasian adult-onset myositis. Ann Rheum Dis. (2009) 68:1621–5. 10.1136/ard.2008.097162 18930994

[30] Allenbach Y Drouot L Rigolet A Charuel JL Jouen F Romero NB. Anti-HMGCR autoantibodies in European patients with autoimmune necrotizing myopathies: inconstant exposure to statin. Medicine. (2014) 93:150–7. 10.1097/MD.0000000000000028 24797170 PMC4632910

[31] Lundberg IE Tjärnlund A Bottai M Werth VP Pilkington C de Visser M. 2017 European League Against Rheumatism/American College of Rheumatology classification criteria for adult and juvenile idiopathic inflammatory myopathies and their major subgroups. Arthritis Rheumatol. (2017) 69:2271–82. 10.1002/art.40320 29106061 PMC5846474

[32] Lundberg IE de Visser M Werth VP. Classification of myositis. Nat Rev Rheumatol. (2018) 14:269–78. 10.1038/nrrheum.2018.41 29651121

[33] Fischer A Antoniou KM Brown KK Cadranel J Corte TJ du Bois RM. An official European Respiratory Society/American Thoracic Society research statement: interstitial pneumonia with autoimmune features. Eur Respir J. (2015) 46:976–87. 10.1183/13993003.00150-2015 26160873

[34] Sambataro G Sambataro D Torrisi SE Vancheri A Pavone M Rosso R. State of the art in interstitial pneumonia with autoimmune features: a systematic review on retrospective studies and suggestions for further advances. Eur Respir Rev. (2018) 27: 10.1183/16000617.0139-2017 29720509 PMC9488748

[35] Chartrand S Swigris JJ Peykova L Chung J Fischer A. A Multidisciplinary evaluation helps identify the antisynthetase syndrome in patients presenting as idiopathic interstitial pneumonia. J Rheumatol. (2016) 43:887–92. 10.3899/jrheum.150966 26932342

[36] Rodero MP Crow YJ. Type I interferon-mediated monogenic autoinflammation: the type I interferonopathies, a conceptual overview. J Exp Med. (2016) 213:2527–38. 10.1084/jem.20161596 27821552 PMC5110029

[37] Horai Y Koga T Fujikawa K Takatani A Nishino A Nakashima Y. Serum interferon-α is a useful biomarker in patients with anti-melanoma differentiation-associated gene 5 (MDA5) antibody-positive dermatomyositis. Mod Rheumatol. (2014) 10.3109/14397595.2014.900843 24716595

[38] Allenbach Y Leroux G Suárez-Calvet X Preusse C Gallardo E Hervier B. Dermatomyositis with or without anti-melanoma differentiation-associated gene 5 antibodies: common interferon signature but distinct NOS2 expression. Am J Pathol. (2016) 186:691–700. 10.1016/j.ajpath.2015.11.010 26806087

[39] Ma W-T Gao F Gu K Chen D-K. The role of monocytes and macrophages in autoimmune diseases: a comprehensive review. Front Immunol. (2019) 10:1140. 10.3389/fimmu.2019.01140 31178867 PMC6543461

[40] Prasse A Pechkovsky DV Toews GB Jungraithmayr W Kollert F Goldmann T. A vicious circle of alveolar macrophages and fibroblasts perpetuates pulmonary fibrosis via CCL18. Am J Respir Crit Care Med. (2006) 173:781–92. 10.1164/rccm.200509-1518OC 16415274

[41] Zhang L Wang Y Wu G Xiong W Gu W Wang C-Y Macrophages: friend or foe in idiopathic pulmonary fibrosis? Respir Res. (2018) 19:170 10.1186/s12931-018-0864-2 30189872 PMC6127991

[42] Horiike Y Suzuki Y Fujisawa T Yasui H Karayama M Hozumi H. Successful classification of macrophage-mannose receptor CD206 in severity of anti-MDA5 antibody positive dermatomyositis associated ILD. Rheumatology. (2019) 58:2143–52. 10.1093/rheumatology/kez185 31143953

[43] Gono T Sato S Kawaguchi Y Kuwana M Hanaoka M Katsumata Y. Anti-MDA5 antibody, ferritin and IL-18 are useful for the evaluation of response to treatment in interstitial lung disease with anti-MDA5 antibody-positive dermatomyositis. Rheumatology. (2012) 51:1563–70. 10.1093/rheumatology/kes102 22589330

[44] Yamada K Asai K Okamoto A Watanabe T Kanazawa H Ohata M. Correlation between disease activity and serum ferritin in clinically amyopathic dermatomyositis with rapidly-progressive interstitial lung disease: a case report. BMC Res Notes. (2018) 11:34. 10.1186/s13104-018-3146-7 29338781 PMC5770999

[45] Gallay L Gayed C Hervier B. Antisynthetase syndrome pathogenesis: knowledge and uncertainties. Curr Opin Rheumatol. (2018) 30:664–73. 10.1097/BOR.0000000000000555 30239350

[46] Chinoy H Adimulam S Marriage F New P Vincze M Zilahi E. Interaction of HLA-DRB1^*^03 and smoking for the development of anti-Jo-1 antibodies in adult idiopathic inflammatory myopathies: a European-wide case study. Ann Rheum Dis. (2012) 71:961–5. 10.1136/annrheumdis-2011-200182 22186711 PMC3371226

[47] Webber MP Moir W Zeig-Owens R Glaser MS Jaber N Hall C. Nested case-control study of selected systemic autoimmune diseases in World Trade Center rescue/recovery workers. Arthritis Rheumatol. (2015) 67:1369–76. 10.1002/art.39059 25779102 PMC5562156

[48] Svensson J Holmqvist M Lundberg IE Arkema EV. Infections and respiratory tract disease as risk factors for idiopathic inflammatory myopathies: a population-based case-control study. Ann Rheum Dis. (2017) 76:1803–8. 10.1136/annrheumdis-2017-211174 28855175

[49] Levine SM Raben N Xie D Askin FB Tuder R Mullins M. Novel conformation of histidyl-transfer RNA synthetase in the lung: the target tissue in Jo-1 autoantibody-associated myositis. Arthritis Rheum. (2007) 56:2729–39. 10.1002/art.22790 17665459

[50] Howard OMZ Dong HF Yang D Raben N Nagaraju K Rosen A. Histidyl-tRNA synthetase and asparaginyl-tRNA synthetase, autoantigens in myositis, activate chemokine receptors on T lymphocytes and immature dendritic cells. J Exp Med. (2002) 196:781–91. 10.1084/jem.20020186 12235211 PMC2194054

[51] Lo W-S Gardiner E Xu Z Lau C-F Wang F Zhou JJ. Human tRNA synthetase catalytic nulls with diverse functions. Science. (2014) 345:328–32. 10.1126/science.1252943 25035493 PMC4188629

[52] Gayed C Uzunhan Y Cremer I Vieillard V Hervier B. Immunopathogenesis of the Anti-Synthetase Syndrome. Crit Rev Immunol. (2018) 38:263–78. 10.1615/CritRevImmunol.2018025744 30806243

[53] Rothwell S Cooper RG Lundberg IE Miller FW Gregersen PK Bowes J. Dense genotyping of immune-related loci in idiopathic inflammatory myopathies confirms HLA alleles as the strongest genetic risk factor and suggests different genetic background for major clinical subgroups. Ann Rheum Dis. (2016) 75:1558–66. 10.1136/annrheumdis-2015-208119 26362759 PMC5300750

[54] Fujisawa T Hozumi H Kono M Enomoto N Hashimoto D Nakamura Y. Prognostic factors for myositis-associated interstitial lung disease. PLoS ONE. (2014) 9:e98824. 10.1371/journal.pone.0098824 24905449 PMC4048238

[55] Tillie-Leblond I Wislez M Valeyre D Crestani B Rabbat A Israel-Biet D. Interstitial lung disease and anti-Jo-1 antibodies: difference between acute and gradual onset. Thorax. (2008) 63:53–9. 10.1136/thx.2006.069237 17557770

[56] Vuillard C Pineton de Chambrun M de Prost N Guérin C Schmidt M Dargent A. Clinical features and outcome of patients with acute respiratory failure revealing anti-synthetase or anti-MDA-5 dermato-pulmonary syndrome: a French multicenter retrospective study. Ann Intensive Care. (2018) 8:87. 10.1186/s13613-018-0433-3 30203297 PMC6131681

[57] Debray M-P Borie R Revel M-P Naccache J-M Khalil A Toper C. Interstitial lung disease in anti-synthetase syndrome: initial and follow-up CT findings. Eur J Radiol. (2015) 84:516–23. 10.1016/j.ejrad.2014.11.026 25541020

[58] Tanizawa K Handa T Nakashima R Kubo T Hosono Y Watanabe K. HRCT features of interstitial lung disease in dermatomyositis with anti-CADM-140 antibody. Respir Med. (2011) 105:1380–7. 10.1016/j.rmed.2011.05.006 21632230

[59] Obert J Freynet O Nunes H Brillet P-Y Miyara M Dhote R. Outcome and prognostic factors in a French cohort of patients with myositis-associated interstitial lung disease. Rheumatol Int. (2016) 36:1727–35. 10.1007/s00296-016-3571-7 27722793

[60] Stanciu R Guiguet M Musset L Touitou D Beigelman C Rigolet A. Antisynthetase syndrome with anti-Jo1 antibodies in 48 patients: pulmonary involvement predicts disease-modifying antirheumatic drug use. J Rheumatol. (2012) 39:1835–9. 10.3899/jrheum.111604 22859355

[61] Travis WD Costabel U Hansell DM King TE Lynch DA Nicholson AG. An official American Thoracic Society/European Respiratory Society statement: Update of the international multidisciplinary classification of the idiopathic interstitial pneumonias. Am J Respir Crit Care Med. (2013) 188:733–48. 10.1164/rccm.201308-1483ST 24032382 PMC5803655

[62] MacDonald SL Rubens MB Hansell DM Copley SJ Desai SR du Bois RM. Nonspecific interstitial pneumonia and usual interstitial pneumonia: comparative appearances at and diagnostic accuracy of thin-section CT. Radiology. (2001) 221:600–5. 10.1148/radiol.2213010158 11719652

[63] Goh NSL Desai SR Veeraraghavan S Hansell DM Copley SJ Maher TM. Interstitial lung disease in systemic sclerosis: a simple staging system. Am J Respir Crit Care Med. (2008) 177:1248–54. 10.1164/rccm.200706-877OC 18369202

[64] Hervier B Meyer A Dieval C Uzunhan Y Devilliers H Launay D. Pulmonary hypertension in antisynthetase syndrome: prevalence, aetiology and survival. Eur Respir J. (2013) 42:1271–82. 10.1183/09031936.00156312 23397301

[65] Bachasson D Dres M Niérat M-C Gennisson J-L Hogrel J-Y Doorduin J. Diaphragm shear modulus reflects transdiaphragmatic pressure during isovolumetric inspiratory efforts and ventilation against inspiratory loading. J Appl Physiol. (2019) 126:699–707. 10.1152/japplphysiol.01060.2018 30730816

[66] Teixeira A Cherin P Demoule A Levy-Soussan M Straus C Verin E. Diaphragmatic dysfunction in patients with idiopathic inflammatory myopathies. Neuromuscul Disord. (2005) 15:32–9. 10.1016/j.nmd.2004.09.006 15639118

[67] Raghu G Remy-Jardin M Myers JL Richeldi L Ryerson CJ Lederer DJ. Diagnosis of Idiopathic Pulmonary Fibrosis. An Official ATS/ERS/JRS/ALAT Clinical Practice Guideline. Am J Respir Crit Care Med. (2018) 198:e44–68. 30168753 10.1164/rccm.201807-1255ST

[68] Marie I Josse S Hatron PY Dominique S Hachulla E Janvresse A. Interstitial lung disease in anti-Jo-1 patients with antisynthetase syndrome. Arthritis Care Res. (2013) 65:800–8. 10.1002/acr.21895 23203765

[69] Marie I Hatron PY Dominique S Cherin P Mouthon L Menard J-F. Short-term and long-term outcomes of interstitial lung disease in polymyositis and dermatomyositis: a series of 107 patients. Arthritis Rheum. (2011) 63:3439–47. 10.1002/art.30513 21702020

[70] Rojas-Serrano J Herrera-Bringas D Mejía M Rivero H Mateos-Toledo H Figueroa JE. Prognostic factors in a cohort of antisynthetase syndrome (ASS): serologic profile is associated with mortality in patients with interstitial lung disease (ILD). Clin Rheumatol. (2015) 34:1563–9. 10.1007/s10067-015-3023-x 26219488

[71] Fathi M Vikgren J Boijsen M Tylen U Jorfeldt L Tornling G. Interstitial lung disease in polymyositis and dermatomyositis: longitudinal evaluation by pulmonary function and radiology. Arthritis Rheum. (2008) 59:677–85. 10.1002/art.23571 18438901

[72] du Bois RM Weycker D Albera C Bradford WZ Costabel U Kartashov A. Six-minute-walk test in idiopathic pulmonary fibrosis: test validation and minimal clinically important difference. Am J Respir Crit Care Med. (2011) 183:1231–7. 10.1164/rccm.201105-0840OC 21131468

[73] Goh NS Hoyles RK Denton CP Hansell DM Renzoni EA Maher TM. Short-term pulmonary function trends are predictive of mortality in interstitial lung disease associated with systemic sclerosis. Arthritis Rheumatol. (2017) 69:1670–8. 10.1002/art.40130 28426895

[74] Trallero-Araguás E Grau-Junyent JM Labirua-Iturburu A García-Hernández FJ Monteagudo-Jiménez M Fraile-Rodriguez G. Clinical manifestations and long-term outcome of anti-Jo1 antisynthetase patients in a large cohort of Spanish patients from the GEAS-IIM group. Semin Arthritis Rheum. (2016) 46:225–31. 10.1016/j.semarthrit.2016.03.011 27139168

[75] Aggarwal R McBurney C Schneider F Yousem SA Gibson KF Lindell K. Myositis-associated usual interstitial pneumonia has a better survival than idiopathic pulmonary fibrosis. Rheumatol. (2017) 56:384–9. 10.1093/rheumatology/kew426 28082622

[76] Cobo-Ibáñez T López-Longo F-J Joven B Carreira PE Muñoz-Fernández S Maldonado-Romero V. Long-term pulmonary outcomes and mortality in idiopathic inflammatory myopathies associated with interstitial lung disease. Clin Rheumatol. (2019) 38:803–15. 10.1007/s10067-018-4353-2 30392161

[77] Fathi M Barbasso Helmers S Lundberg IE. KL-6: a serological biomarker for interstitial lung disease in patients with polymyositis and dermatomyositis. J Intern Med. (2012) 271:589–97. 10.1111/j.1365-2796.2011.02459.x 21950266

[78] Koenig M Fritzler MJ Targoff IN Troyanov Y Senécal J-L. Heterogeneity of autoantibodies in 100 patients with autoimmune myositis: insights into clinical features and outcomes. Arthritis Res Ther. (2007) 9:R78. 10.1186/ar2276 17688695 PMC2206383

[79] La Corte R Lo Mo Naco A Locaputo A Dolzani F Trotta F. In patients with antisynthetase syndrome the occurrence of anti-Ro/SSA antibodies causes a more severe interstitial lung disease. Autoimmunity. (2006) 39:249–53. 10.1080/08916930600623791 16769659

[80] Le Goff B Chérin P Cantagrel A Gayraud M Hachulla E Laborde F. Pneumomediastinum in interstitial lung disease associated with dermatomyositis and polymyositis. Arthritis Rheum. (2009) 61:108–18. 10.1002/art.24372 19116970

[81] Selva-O'Callaghan A Labrador-Horrillo M Muñoz-Gall X Martínez-Gomez X Majó-Masferrer J Solans-Laque R. Polymyositis/dermatomyositis-associated lung disease: analysis of a series of 81 patients. Lupus. (2005) 14:534–42. 10.1191/0961203305lu2158oa 16130510

[82] Machuca JS Cos J Niazi M Fuentes G-D. Spontaneous pneumomediastinum in a patient with facial rash. J Bronchol Interv Pulmonol. (2010) 17:59–63. 10.1097/LBR.0b013e3181ca6b6c 23168662

[83] Powell C Kendall B Wernick R Heffner JE. A 34-year-old man with amyopathic dermatomyositis and rapidly progressive dyspnea with facial swelling. Diagnosis: pneumomediastinum and subcutaneous emphysema secondary to amyopathic dermatomyositis-associated interstitial lung disease. Chest. (2007) 132:1710–3. 10.1378/chest.07-0286 17998377

[84] Ma X Chen Z Hu W Guo Z Wang Y Kuwana M. Clinical and serological features of patients with dermatomyositis complicated by spontaneous pneumomediastinum. Clin Rheumatol. (2016) 35:489–93. 10.1007/s10067-015-3001-3 26149923

[85] Kuronuma K Honda H Mikami T Saito A Ikeda K Otsuka M. Response to pneumococcal vaccine in interstitial lung disease patients: influence of systemic immunosuppressive treatment. Vaccine. (2018) 36:4968–72. 10.1016/j.vaccine.2018.06.062 29983256

[86] Redondo-Benito A Curran A Villar-Gomez A Trallero-Araguas E Fernández-Codina A Pinal-Fernandez I. Opportunistic infections in patients with idiopathic inflammatory myopathies. Int J Rheum Dis. (2018) 21:487–96. 10.1111/1756-185X.13255 29314762 PMC11669102

[87] Park JW Curtis JR Moon J Song YW Kim S Lee EB. Prophylactic effect of trimethoprim-sulfamethoxazole for pneumocystis pneumonia in patients with rheumatic diseases exposed to prolonged high-dose glucocorticoids. Ann Rheum Dis. (2018) 77:644–9. 10.1136/annrheumdis-2017-211796 29092853 PMC5909751

[88] Dowman LM McDonald CF Hill CJ Lee AL Barker K Boote C. The evidence of benefits of exercise training in interstitial lung disease: a randomised controlled trial. Thorax. (2017) 72:610–9. 10.1136/thoraxjnl-2016-208638 28213592

[89] Zisman DA Kawut SM Lederer DJ Belperio JA Lynch JP Schwarz MI. Serum albumin concentration and waiting list mortality in idiopathic interstitial pneumonia. Chest. (2009) 135:929–35. 10.1378/chest.08-0754 19017875 PMC2666778

[90] Troyanov Y Targoff IN Tremblay J-L Goulet J-R Raymond Y Senécal J-L. Novel classification of idiopathic inflammatory myopathies based on overlap syndrome features and autoantibodies: analysis of 100 French Canadian patients. Medicine. (2005) 84:231–49. 10.1097/01.md.0000173991.74008.b0 16010208

[91] Wilkes MR Sereika SM Fertig N Lucas MR Oddis CV. Treatment of antisynthetase-associated interstitial lung disease with tacrolimus. Arthritis Rheum. (2005) 52:2439–46. 10.1002/art.21240 16052580

[92] Yamasaki Y Yamada H Yamasaki M Ohkubo M Azuma K Matsuoka S. Intravenous cyclophosphamide therapy for progressive interstitial pneumonia in patients with polymyositis/dermatomyositis. Rheumatology. (2007) 46:124–30. 10.1093/rheumatology/kel112 16754626

[93] Sharma N Putman MS Vij R Strek ME Dua A. Myositis-associated interstitial lung disease: predictors of failure of conventional treatment and response to tacrolimus in a US cohort. J Rheumatol. (2017) 44:1612–8. 10.3899/jrheum.161217 28864644

[94] Marie I Hatron P-Y Cherin P Hachulla E Diot E Vittecoq O. Functional outcome and prognostic factors in anti-Jo1 patients with antisynthetase syndrome. Arthritis Res Ther. (2013) 15:R149. 10.1186/ar4332 24286268 PMC3978997

[95] Douglas WW Tazelaar HD Hartman TE Hartman RP Decker PA Schroeder DR. Polymyositis-dermatomyositis-associated interstitial lung disease. Am J Respir Crit Care Med. (2001) 164:1182–5. 10.1164/ajrccm.164.7.2103110 11673206

[96] Huapaya JA Silhan L Pinal-Fernandez I Casal-Dominguez M Johnson C Albayda J. Long-term treatment with azathioprine and mycophenolate mofetil for myositis-related interstitial lung disease. Chest. (2019) 10.1016/j.chest.2019.05.023 31238042 PMC6945652

[97] Mira-Avendano IC Parambil JG Yadav R Arrossi V Xu M Chapman JT. A retrospective review of clinical features and treatment outcomes in steroid-resistant interstitial lung disease from polymyositis/dermatomyositis. Respir Med. (2013) 107:890–6. 10.1016/j.rmed.2013.02.015 23517887

[98] Kurita T Yasuda S Oba K Odani T Kono M Otomo K The efficacy of tacrolimus in patients with interstitial lung diseases complicated with polymyositis or dermatomyositis. Rheumatol. (2015) 54:39–44. 10.1093/rheumatology/keu166 24764266

[99] Huapaya JA Hallowell R Silhan L Pinal-Fernandez I Casal-Dominguez M Johnson C. Long-term treatment with human immunoglobulin for antisynthetase syndrome-associated interstitial lung disease. Respir Med. (2019) 154:6–11. 10.1016/j.rmed.2019.05.012 31176796 PMC11678789

[100] Allenbach Y Guiguet M Rigolet A Marie I Hachulla E Drouot L. Efficacy of Rituximab in Refractory Inflammatory Myopathies Associated with Anti- Synthetase Auto-Antibodies: An Open-Label, Phase II Trial. PLoS ONE. (2015) 10:e0133702. 10.1371/journal.pone.0133702 26539981 PMC4634756

[101] Andersson H Sem M Lund MB Aaløkken TM Günther A Walle-Hansen R. Long-term experience with rituximab in anti-synthetase syndrome-related interstitial lung disease. Rheumatol. (2015) 54:1420–8. 10.1093/rheumatology/kev004 25740830

[102] Sharp C McCabe M Dodds N Edey A Mayers L Adamali H Rituximab in autoimmune connective tissue disease-associated interstitial lung disease. Rheumatol. (2016) 55:1318–24. 10.1093/rheumatology/kew195 27060110

[103] Doyle TJ Dhillon N Madan R Cabral F Fletcher EA Koontz DC. Rituximab in the treatment of interstitial lung disease associated with antisynthetase syndrome: a multicenter retrospective case review. J Rheumatol. (2018) 45:841–50. 10.3899/jrheum.170541 29606668 PMC5984657

[104] Fasano S Gordon P Hajji R Loyo E Isenberg DA. Rituximab in the treatment of inflammatory myopathies: a review. Rheumatol. (2017) 56:26–36. 10.1093/rheumatology/kew146 27121778

[105] Zou J Li T Huang X Chen S Guo Q Bao C. Basiliximab may improve the survival rate of rapidly progressive interstitial pneumonia in patients with clinically amyopathic dermatomyositis with anti-MDA5 antibody. Ann Rheum Dis. (2014) 73:1591–3. 10.1136/annrheumdis-2014-205278 24739327

[106] Chen Z Wang X Ye S. Tofacitinib in amyopathic dermatomyositis-associated interstitial lung disease. N Engl J Med. (2019) 381:291–3. 10.1056/NEJMc1900045 31314977

[107] Dastmalchi M Grundtman C Alexanderson H Mavragani CP Einarsdottir H Helmers SB. A high incidence of disease flares in an open pilot study of infliximab in patients with refractory inflammatory myopathies. Ann Rheum Dis. (2008) 67:1670–7. 10.1136/ard.2007.077974 18272672

[108] Distler O Highland KB Gahlemann M Azuma A Fischer A Mayes MD. Nintedanib for systemic sclerosis-associated interstitial lung disease. N Engl J Med. (2019) 380:2518–28. 10.1056/NEJMoa1903076 31112379

[109] Courtwright AM El-Chemaly S Dellaripa PF Goldberg HJ. Survival and outcomes after lung transplantation for non-scleroderma connective tissue-related interstitial lung disease. J Heart Lung Transplant. (2017) 36:763–9. 10.1016/j.healun.2016.12.013 28131664

[110] Ameye H Ruttens D Benveniste O Verleden GM Wuyts WA. Is lung transplantation a valuable therapeutic option for patients with pulmonary polymyositis? Experiences from the Leuven transplant cohort. Transplant Proc. (2014) 46:3147–53. 10.1016/j.transproceed.2014.09.163 25420846

